# Response to letter re: Carotid atherosclerotic plaques standardized uptake values: methodological issues on reproducibility and accuracy

**DOI:** 10.1186/s13550-017-0309-9

**Published:** 2017-09-07

**Authors:** Nicola Giannotti, Martin J. O’Connell, Shane J. Foley, Marie C. Galligan, Peter J. Kelly, Jonathan P. McNulty

**Affiliations:** 1 Radiography and Diagnostic Imaging, School of Medicine, University College Dublin, Dublin, Ireland; 2 Department of Radiology, Mater Misericordiae University Hospital, Dublin, Ireland; 3 School of Medicine, University College Dublin, Dublin, Ireland; 4 UCD Clinical Research Centre, School of Medicine, University College Dublin, Dublin, Ireland; 5 Neurovascular Clinical Science Unit, Stroke Service and Department of Neurology, Mater University Hospital, Dublin, Ireland

## Background

Dear Editor,

We would like to thank Dr. Siamak Sabour for his letter and comments relating to our recently published article [1]. In the manuscript, an investigation was conducted on whether or not carotid atherosclerotic plaque standardized uptake values (SUVs) are consistent and reproducible across software packages; therefore, the purpose of the analysis performed was to measure the reproducibility, rather than validity, of SUV measurements between two software packages (OsiriX MD® version 6.5.2, Pixmeo© SARL, Geneva, Switzerland and AquariusNet iNtuitionTM version 4.4.11, TeraRecon, Foster City, CA, USA) (Table 1).

**Table 1:** SUV measurements (mean and standard deviation) by location and software together with differences (mean and standard deviation)

Common carotid (CC) and internal carotid (IC) arteries	TeraRecon values (mean ± SD)	Osirix values (mean ± SD)	Difference in values (mean ± SD)
SUV mean bifurcation left	1.5 **±** 0.36	1.58 ± 0.43	0.08 ± 0.003
SUV mean bifurcation right	1.56 **±** 0.38	1.67 ± 0.46	0.11 ± 0.006
SUV mean CC left	1.08 **±** 0.59	1.17 ± 0.6	0.09 ± 0.004
SUV mean CC right	1.11 **±** 0.61	1.23 ± 0.64	0.12 ± 0.007
SUV mean IC left	1.56 **±** 0.45	1.71 ± 0.57	0.15 ± 0.01
SUV mean IC right	1.66 **±** 0.38	1.72 ± 0.44	0.06 ± 0.002
SUV max bifurcation left	2.54 **±** 0.65	2.5 ± 0.7	0.04 ± 0.008
SUV max bifurcation right	2.6 **±** 0.69	2.62 ± 0.73	0.02 ± 0.002
SUV max CC left	2.09 **±** 0.91	2.06 ± 0.88	0.03 ± 0.001
SUV max CC right	2.14 **±** 0.97	2.16 ± 0.99	0.02 ± 0.001
SUV max IC left	2.52 **±** 0.67	2.57 ± 0.84	0.05 ± 0.001
SUV max IC right	2.6 **±** 0.69	2.58 ± 0.74	0.02 ± 0.001
SUV min bifurcation left	0.71 **±** 0.3	0.87 ± 0.36	0.16 ± 0.012
SUV min bifurcation right	0.76 **±** 0.3	0.94 ± 0.38	0.18 ± 0.016
SUV min CC left	0.45 **±** 0.36	0.56 ± 0.46	0.11 ± 0.006
SUV min CC right	0.48 **±** 0.39	0.62 ± 0.48	0.14 ± 0.01
SUV min IC left	0.86 **±** 0.34	1 ± 0.43	0.14 ± 0.01
SUV min IC right	0.84 **±** 0.29	1 ± 0.34	0.16 ± 0.012

## Conclusions

We acknowledge that the *p* values reported in the manuscript previously submitted are dependent on the study sample size, and may not provide sufficient support of measurement reliability. Thus, we will now provide the intra-class coefficient (ICC) for the relevant variables (see Tables 2, 3, and 4) which was found to be supportive of our initial findings.

**Table 2:** ICC for SUVs mean with 95% confidence intervals

Location	ICC	Confidence limit minimum	Confidence limit maximum
SUV mean bifurcation left	0.843	0.806	0.872
SUV mean bifurcation right	0.787	0.733	0.828
SUV mean CC left	0.906	0.887	0.922
SUV mean CC right	0.924	0.858	0.924
SUV mean IC left	0.79	0.713	0.842
SUV mean IC right	0.828	0.727	0.883

**Table 3:** ICC for SUVs max with 95% confidence intervals

Location	ICC	Confidence limit minimum	Confidence limit maximum
SUV max bifurcation left	0.826	0.803	0.846
SUV max bifurcation right	0.817	0.793	0.838
SUV max CC left	0.83	0.808	0.85
SUV max CC right	0.891	0.803	0.891
SUV max IC left	0.752	0.699	0.795
SUV max IC right	0.791	0.721	0.838

**Table 4:** ICC for SUVs min with 95% confidence intervals

Location	ICC	Confidence limit minimum	Confidence limit maximum
SUV min bifurcation left	0.627	0.494	0.773
SUV min bifurcation right	0.635	0.434	0.748
SUV min CC left	0.74	0.694	0.783
SUV min CC right	0.65	0.545	0.705
SUV min IC left	0.788	0.656	0.858
SUV min IC right	0.428	0.27	0.546

As expected, higher agreements (ICC) were found among SUV mean and maximum measurements. Effect size measurements also show that SUV max measurements were similar when compared (differences in mean values within the range: 0.02–0.05).

Once again we thank Dr. Sabour for his contribution to the important discussion around SUV measurements across software packages.
